# Identification of Differentially Expressed Genes in the Hypothalamus of Broilers Under Heat Stress Using Transcriptome Analysis

**DOI:** 10.3390/ani15040502

**Published:** 2025-02-10

**Authors:** Xiaofang He, Bingbing Ma, Lin Zhang, Feng Gao

**Affiliations:** 1 Jinling Institute of Science and Technology, College of Animal Science and Food Engineering, No. 99, Hongjing Avenue, Jiangning District, Nanjing 211169, China; hexiaofang@jit.edu.cn; 2 College of Animal Science and Technology, Nanjing Agricultural University, No. 1 Weigang, Nanjing 210095, China; zhanglin2012@njau.edu.cn; 3 College of Smart Animal Husbandry, College of Biology and Food, Shangqiu Normal University, Shangqiu 476000, China; mabingbing3062@163.com

**Keywords:** broiler, heat stress, hypothalamus, transcriptome analysis

## Abstract

The hypothalamus is the advanced center that regulates visceral activities under the cerebral cortex. It plays some key roles, such as regulating body temperature, assessing feed intake, and balancing blood glucose and endocrine gland activities. Heat stress is known to trigger a series of detrimental consequences, prominently featuring a reduction in feed intake, an elevation in body temperature, and other related phenomena. To understand the mechanisms of how heat stress affects the function of the hypothalamus, broilers were allocated to three groups: the normal control (NC) group, the heat-stress (HS) group, and the pair-fed (PF) group. The PF group was established with the aim of eliminating the confounding effect of reduced feed intake. The trial lasted for two weeks, from the age of 28 to 42 d. A total of 280 differential expressed genes (DEGs) were identified (*p*_adj_ < 0.05, |log_2_(FC)| ≥ 1) among three groups, including 3 up-regulated and 112 down-regulated genes in the HS group compared to the NC group, and 3 up-regulated and 13 down-regulated genes between the PF and NC groups. Compared with the HS group, a total of 149 genes were identified in the PF group, of which 125 genes were up-regulated and 24 genes were down-regulated. Gene Ontology enrichment indicated that a subset of DEGs was involved in brain development, the central nervous system (CNS), nerve signal transduction, and calcium homeostasis. The solute carrier family 1 member A6 and solute carrier family 6 member 13, identified as down-regulated genes (*p*_adj_ < 0.05) in the HS group, were considered as key genes in Gamma-aminobutyric acid (GABA) transportation, the normal expression of which ensures that extracellular GABA is maintained at a certain level and provides the amino acids needed for metabolism. Simultaneously, the solute carrier family 13 member 4 and solute carrier family 16 member 8 were also identified as down-regulated, which indicated that heat stress resulted in disorder and physiologic derangement in the hypothalamus. Meanwhile, the anorexigenic part of pro-opiomelanocortin genes was up-regulated significantly in the HS group. The transcriptome sequencing results can help us understand the regulatory mechanism of feed intake decline in broilers under heat stress at the genetic level.

## Simple Summary

The hypothalamus is the advanced center that regulates visceral activities under the cerebral cortex. It plays some key roles, such as regulating appetite. Heat stress can induce many unfavorable effects, including reduced feed intake, increased body temperature and others. To understand the mechanisms of how heat stress affects the function of the hypothalamus, broilers were allocated to three groups: the normal control (NC) group, heat-stress (HS) group, and pair-fed (PF) group, from the age of 28 to 42 d. A total of 280 differential expressed genes were identified. Gene Ontology enrichment showed relevant involvement. The key genes related to GABA transportation, alongside others, were down-regulated in the HS group, and the anorexigenic component of the pro-opiomelanocortin gene was up-regulated. The transcriptome sequencing results clarify the role of genetic regulatory mechanisms in the decline of feed intake under heat stress. This can assist in the formulation of measures to reduce the adverse effects of heat stress on broiler production. Such measures are of great value to the poultry industry in terms of optimizing and upgrading feed formulas.

## 1. Introduction

The overall trend of chicken production and consumption has been increasing gradually in recent years, and China has become the second-largest country for chicken production and consumption in the world. At present, the number of fast-growing commercial broilers in tropical and subtropical regions is gradually increasing, and the heat stress induced by high temperatures has become the main factor restricting the development of the broiler industry, along with the high-density and large-scale feeding mode [1]. Studies have shown that heat stress leads to decreased appetite, less feed intake, altered nutrient metabolism pathways, and, ultimately, the reduced growth performance of broilers [2,3]. Our previous study found that heat stress significantly reduced appetite and average daily feed intake in broilers, decreased the nutrient utilization rate of feed, and finally depressed growth performance [4]. Traditional studies often focus on the digestion and absorption of feed under heat stress in broilers. In recent years, various high-throughput sequencing technologies have been used to identify differential genes based on transcriptome sequencing in poultry research. Finally, studies have elucidated the mechanism of appetite loss in broilers.

The hypothalamus, which is an important part of the CNS, plays a key role in regulating feed intake, maintaining body temperature and endocrines through different pathways, as well as its involvement with emergency responses to stressors in the environment [5,6]. This small yet highly specialized brain region integrates a multitude of signals from both internal and external sources to precisely modulate appetite and maintain energy homeostasis [7,8,9]. The formation of appetite is very complex. The hypothalamus is the main integration center of various appetite regulation signals, which mainly include two opposite branches of the melanocortin signaling pathways, namely the orexigenic neuron neuropeptide/agouti-related protein (NPY/AgRP), as well as the anorexigenic neuron of pro-opiomelanocortin and cocaine/amphetamine-regulated transcription peptide (*POMC*/*CART*). Furthermore, it is responsible for forming feelings of hunger and satiety, ultimately regulating feed intake and energy expenditure [9,10]. Our previous study demonstrated that the expression of NPY in the hypothalamus of broilers was down-regulated under heat stress conditions [11]. Pro-opiomelanocortin (*POMC*) is a precursor polypeptide, where the *POMC* gene is transcribed and translated into a large precursor protein. In the hypothalamus, the *POMC* gene is under the tight regulation of various factors, including hormones and nutrients [12]. The *POMC* precursor is then cleaved by specific proteases into several biologically active peptides. In the hypothalamus, the main cleavage products relevant to appetite regulation are the α-melanocyte-stimulating hormone (α-MSH) and β-endorphin [13].

RNA-sequencing (RNA-seq), also known as transcriptome sequencing, has been utilized widely in animal science technology developed for transcriptome analysis using new-generation sequencing technology, which can comprehensively and rapidly obtain the sequence information and expression information of almost all transcripts of a specific cell or tissue in a certain state. Including protein-coding mRNAs and various non-coding RNAs, alternative gene splicing produces different amounts of transcript expression [14]. In brief, this technology is expected to provide novel insight into the effects of heat stress on the hypothalamus of broilers. Previously, we found that heat stress reduced feed intake and altered the gene expression of appetite-related hormones in the intestinal tract of broilers [4]. However, the molecular mechanism of heat stress in the hypothalamus remains scarce. Therefore, it is critical to reveal the heat-stress-mediated mechanisms of reducing appetite and impairing nutrient absorption for animal nutritionists. The objective of this study was to identify differentially expressed genes in the hypothalamus of broilers under heat stress based on the transcriptional level. These results provide a new perspective regarding the regulation of appetite in commercial broilers while offering a basis for maintaining their growth performance.

## 2. Materials and Methods

### 2.1. Broilers’ Management and Sample Collection

Two hundred newly hatched male Arbor Acres broilers were obtained from a commercial hatchery and raised in battery brooders. From 1 d to 27 d of age, the birds received commercial standard diets and management.

At 28 d of age, a total of 144 chicks with similar body weights (1.10 ± 0.02 kg) were selected and equally distributed into three groups with 6 cages per group and 8 chickens per cage. In the normal control (NC) group, chickens were kept at 22 °C ambient temperature and provided with ad libitum access to feed; in the heat stress (HS) group, chickens were reared at a 32 °C successive temperature, and provided with ad libitum access to feed; and in the pair-fed (PF) group, chickens were maintained at 22 °C and received the amount of feed each day equal to the feed consumed in previous day by the HS group in order to avoid the effects of different nutritional levels on the calculation of heat tolerance. On each day, the average feed intake per chicken was measured in the HS group. This exact amount was offered to the chickens in the PF group on the next day. The PF group was designed to understand the effects of heat stress on the gene expressions in the hypothalamus independent of a reduction in feed intake. For all three groups, the relative humidity was maintained at 55 ± 5%. All birds received common commercial grower feed, and water was provided for ad libitum consumption. Lighting was continuously provided throughout the experimental period.

After 14 days of heat stress exposure, two broilers with weights close to the average weight of the replicate were randomly selected from each cage. These broilers were slaughtered through exsanguination without prior fasting. The heads of the broilers were carefully dissected to isolate the entire hypothalamus. These samples were then immediately stored at −80 °C for subsequent analysis.

### 2.2. Ethical Statement

All animal experiments were carried out in compliance with the Guidelines for the Care and Use of Laboratory Animals, prepared by the Institutional Animal Welfare and Ethics Committee of Nanjing Agricultural University, Nanjing, China [certification no: SYXK (Su) 2022-0031].

### 2.3. Library Construction and Sequencing of the Transcriptome

Total RNA was isolated from the frozen hypothalamus of the three groups using the TRIzol reagent (Invitrogen, Carlsbad, CA, USA). The quantity and quality of the RNA were determined using the NanoDrop spectrophotometer (GE Healthcare Life Sciences, Uppsala, Sweden), Qubit^®^ RNA Assay Kit in Qubit^®^ 2.0 Fluorometer (Life Technologies, Gaithersburg, MD, USA), and RNA Nano 6000 Assay Kit of the Bioanalyzer 2100 system (Agilent Technologies, Santa Clara, CA, USA). For each RNA-seq sample, total hypothalamus RNA consisted of a mixture of every four broilers. Sequencing libraries were generated using NEBNext^®^ Ultra^TM^ RNA Library Prep Kit for Illumina^®^ (New England Biolabs, Beverly, MA, USA) following the manufacturer’s recommendations. Briefly, mRNA was extracted from total RNA using oligo (dT) magnetic beads (TaKaRa, Osaka, Japan) and sheared into short fragments of about 200 bases. We rapidly reverse-transcribed the RNA samples with qualified concentrations using the PrimeScript^TM^ RT Master Mix reverse transcription kit according to their instruction. The cDNA library (PE 150) was sequenced using an Illumina HiSeq^TM^ 2500 platform.

### 2.4. Data Analysis of the Transcriptome

The Illumina raw reads were quality-controlled by removing low-quality sequences (more than half of the base qualities less than 5) or reads with more than 10% unknown nucleotides (N) and sequencing adapters. Clean reads were aligned with the chicken genome (GRCg6a) through TopHat software2.1.1, allowing less than 2 mismatches. Unmapped and multi-position matched reads were excluded from further analyses. The gene expression level was calculated using the FPKM (Fragments per Kilobase per Millon Mapped Fragments) method [15], and the differential expression analysis among three groups was performed using the DESeq2 package [16]. Genes with an adjusted *p*-value (*p*_adj_) < 0.05 and the absolute value of |log_2_ (fold change)| > 1 were assigned as DEGs. The functional enrichment of differential genes by GO and KEGG was performed using the cluster Profiler package. We utilized the R package cluster Profiler to analyze GO and KEGG pathway enrichment [17]. The GO terms and pathways, as well as KEGG pathways with q values (adjusted *p*-value by BH method) <0.05, were considered to be significantly enriched ones.

### 2.5. Validations by qPCR

Seven genes were selected to verify the DEG results. RNA samples from the six animals for RNA-Seq were analyzed by quantitative real-time PCR (ABI 7500 Real-Time PCR). The reverse transcription was performed using PrimeScript RT Master Mix (TaKaRa, Osaka, Japan) according to the manufacturer’s instructions. qPCR was performed with FastStart Universal SYBR Green Master (ROX) (Roche Diagnostics, Indianapolis, IN, USA). The primers used for qPCR are listed in Table S1. All reactions were run in duplicate, and data were normalized using the housekeeping gene *GAPDH*. Each gene in every sample was replicated three times, and then the average Ct value was obtained. The relative expression level of each gene corresponding to each sample was calculated according to the method of Livak and Schmittgen (2001) [18]. The *t*-test was used to compare the levels of expression between the HS and NC groups, and the significance level was set to *p*-value < 0.05.

## 3. Results

### 3.1. Transcriptome Sequencing Data and Quality Control

**Figure 1:**
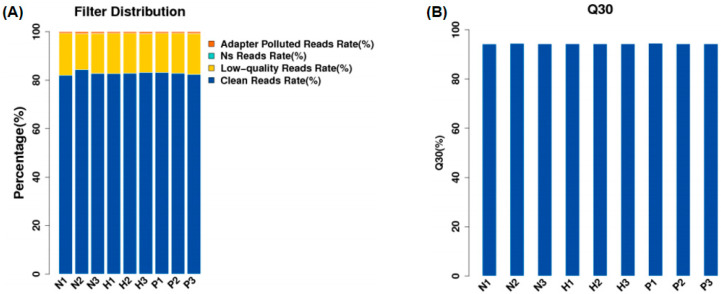
Filter distribution (**A**) and Q30 quality control (**B**). (NC group: N1, N2, N3; HS group: H1, H2, H3; PF group: P1, P2, P3).

As shown in Supplementary Table S2, a total of 477 million raw reads were generated from the nine samples. After quality control, 349 million clean reads were obtained, and the clean reads rate of all samples was more than 80% (Figure 1A). The Q30 values of clean reads for all nine samples were greater than 90% (Figure 1B).

**Figure 2:**
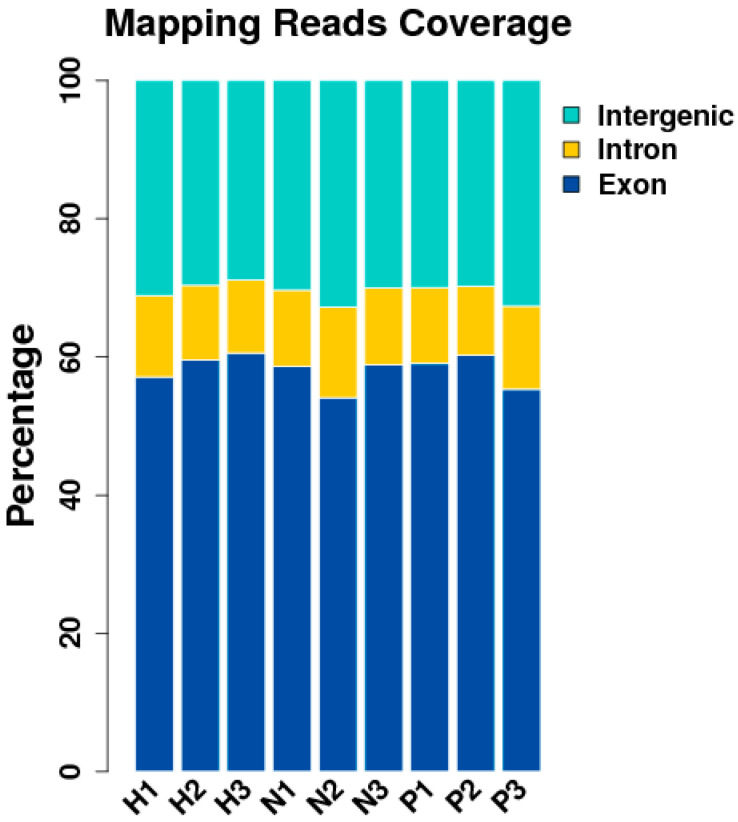
Distribution of unique alignment sequences in each region of the reference genome gene. (NC group: N1, N2, N3; HS group: H1, H2, H3; PF group: P1, P2, P3).

Some of the original sequences obtained by sequencing contained sequencing adapter sequences and low-quality sequences to ensure that the data were analyzed. We filtered the original sequence to obtain high-quality clean reads, which were then followed by analysis and subsequent analysis for clean reads (Figure 2).

**Figure 3:**
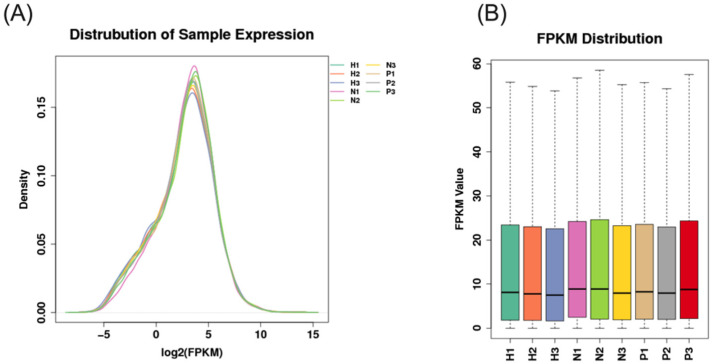
Distribution of nine samples of expression ((**A**), distribution of samples expression; (**B**), FPKM distribution. NC group: N1, N2, N3; HS group: H1, H2, H3; PF group: P1, P2, P3).

After quality control, Tophat software was employed to align the 51 to 60 million (M) clean reads onto the reference chicken genome (GRCg6a) with a tolerance of 2 bp mismatches [19]. About 79.66 to 92.90% of reads were mapped to the chicken reference genome, of which 71.90 to 78.50% were located in annotated exons, 6.80 to 10.30% were assigned to introns, and the remaining 14.60 to 17.70% fell in intergenic regions (GRCg6a) (Figure 3).

**Figure 4:**
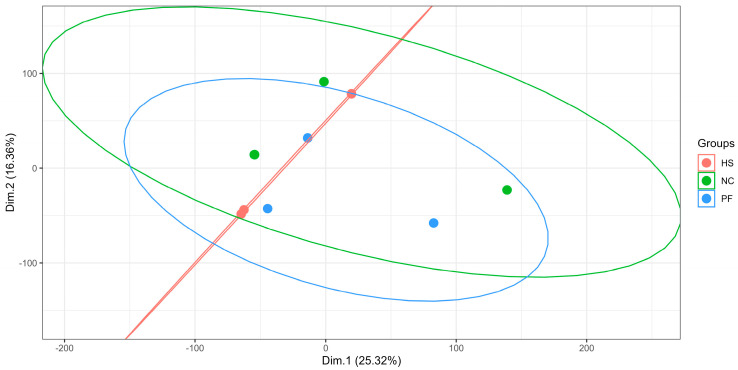
The PCA of three groups (NC, normal control group; HS, heat stress group; PF, pair feed group. Dim.1, dimension1; Dim.2, dimension2).

As shown in Figure 4, the PCA among the three groups had no significant difference.

### 3.2. Differentially Expressed Genes Among Three Groups

**Figure 5:**
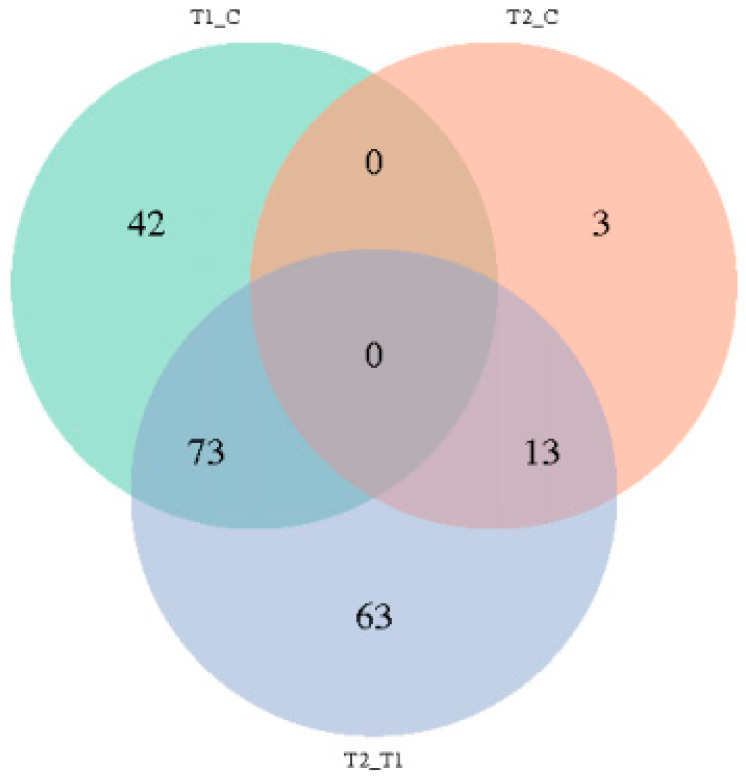
The Venn diagram of differentially expressed genes among the three groups. (T1, heat stress group; T2, pair feed group; C, normal control group).

As shown in Table 1 and Figure 5, a total of 280 different genes were identified among the three groups. A total of 115 genes were identified in the HS compared with the NC group, among which 3 genes were up-regulated, and 112 genes were down-regulated. Compared with NC, a total of 16 genes were identified, including 3 up-regulated genes and 13 down-regulated genes in the PF group. Compared with the PF group, a total of 149 genes were identified in the HS group, including 24 up-regulated genes and 125 down-regulated genes.

### 3.3. Functional Enrichment Analysis of the DEGs

**Figure 6:**
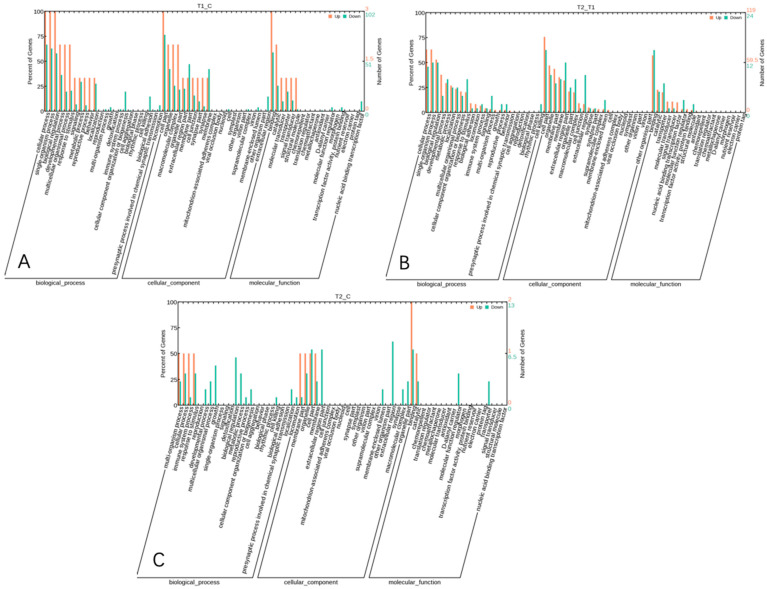
GO statistical bar chart of differentially expressed gene clusters among the three groups ((**A**), HS vs. NC; (**B**), HS vs. PF; (**C**), PF vs. NC).

GO provides a systematic classification and comprehensive description of the attributes of genes and their products [20]. GO enrichment analysis was performed for 280 differentially expressed genes. A total of 128 significantly enriched GO terms were identified between the HS and the NC group, involving 3 physiological and biochemical processes, including 59 biological processes (BP), 27 cellular components, CC), and 42 molecular functions (MF) (Figure 6A).

A total of 127 significantly enriched GO terms were identified between the PF group and HS group, including 61 BP, 25 CC, and 41 MF terms (Figure 6B).

A total of 87 significantly enriched GO terms were identified between the PF group and NC group, including 51 BP, 17 CC, and 19 MF terms (Figure 6C).

Enrichment of the KEGG pathway by differential genes of the HS and NC group resulted in the enrichment of pathway protein digestion and absorption.

### 3.4. qPCR Analysis

**Figure 7:**
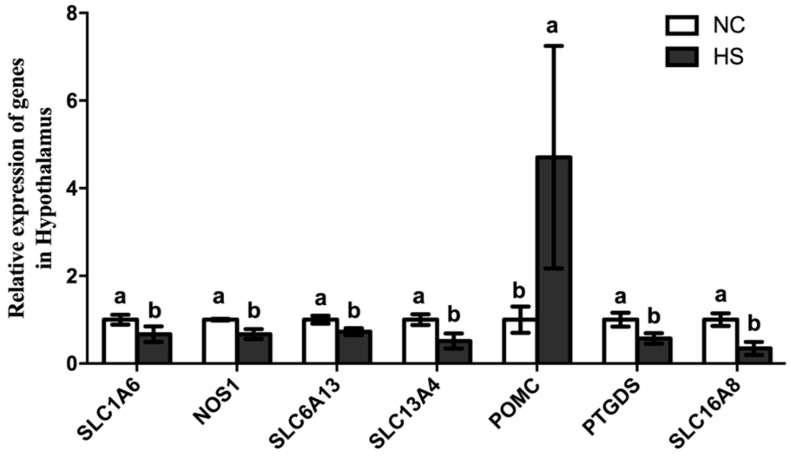
The expression of differentially expressed genes in qPCR between the HS and NC groups. Note: a,b represents a significant difference in the expression of the genes between the HS and NC groups (*p* < 0.05). *SLC1A6*, solute carrier family 1 member 6; *NOS1*, nitric oxide synthase 1; *SLC6A13*, solute carrier family 6 member 13; *SLC13A4*, solute carrier family 13 member 4; *POMC*, pro-opiomelanocortin; *PTGDS* stands for prostaglandin D2 synthase; *SLC16A8*, solute carrier family 16 member 8.

In order to validate the results from RNA-Seq, seven DEGs were selected for relative quantitative analysis between the HS and NC groups (Table 2). The selected DEGs included one up-regulated DEG (*POMC*) and six down-regulated DEGs (*SLC1A6*, *SLC6A13*, *SLC13A4*, *SLC16A8*, *PTGDS*, and *NOS1*). As shown in Figure 7, qPCR validated the results of the DGE analysis in all cases. The investigation of candidate genes for appetite and the CNS was performed through the integrated analysis of QTL detection and differential gene expressions.

## 4. Discussion

This experiment utilized RNA sequencing technology to identify the influence of heat stress on genetic variations. In contrast, it was found that the top ten most significantly enriched GO terms showed differences mainly in anion transport, chemical synaptic transmission, cell cation homeostasis, CNS development, cell ion-related processes, cell signal transduction, cell adhesion, brain development, and cell calcium ion homeostasis. In the present study, among the 280 DEGs obtained, they were predominantly implicated in biological processes such as sodium ion transport, cell membrane transport, and calcium ion homeostasis. This finding suggests that heat stress may have an impact on ion transport, which, in turn, disrupts the body’s homeostasis. Several genes were involved in neuronal differentiation, amino acid transport, galanin receptors, nitric oxide synthase, sodium–chlorine-dependent transporters, appetite regulation gene *POMC*, and lipid transport. Among the seven DEGs verified, *SLC1A6* and *SLC6A13* were both GABA transporters. The SLC family consists of over 400 transporter proteins, which are classified into 65 sub-families based on their sequence homology and substrate specificity. These transporters are widely distributed across different cell types and tissues, playing crucial roles in various physiological processes [21]. *SLC1A6* contributes to the maintenance of intracellular and extracellular amino acid balance. Glutamate and other amino acids transported by *SLC1A6* are not only neurotransmitters but also important metabolites. They are involved in protein synthesis, energy metabolism, and the synthesis of other biologically active molecules. The physiological expression level of *SLC1A6* can make extracellular GAB maintain a certain level and provide amino acids for metabolism [22,23,24]. *SLC6A13*, also known as the GABA transporter 4, plays a key role in maintaining the balance between excitatory and inhibitory neurotransmission in the brain. The dysregulation of the *SLC6A13* function has been associated with various neurological disorders [23]. The SLC16 gene family consists of 14 members, also known as the monocarboxylic acid transporter (MCT) family, which are involved in a wide range of metabolic pathways, energy metabolism, gluconeogenesis, T lymphocyte activation, intestinal metabolism, spermatogenesis, pancreatic β cell dysfunction, thyroid hormone metabolism and drug transport in the brain, skeletal muscle, heart, and tumor cells [25]. These transport processes are proton-linked, electrically neutral, and driven by the concentration gradient of the substrate. MCT1, MCT2, and MCT4 were widely expressed in various tissues. Meanwhile, the dysfunction of MCT has been linked to devastating diseases such as obesity and ischemia. Heat stress often induces blood flow to the surface of broilers, leading to intestine ischemia and further changes in the status of ion transportation [26]. *SLC16A8* is mainly responsible for the transport of monocarboxylic acid metabolites to maintain cellular homeostasis (e.g., pyruvate, L-lactic acid, and ketone bodies) [27]. Study has demonstrated that *SLC16A* expression is abnormal under the condition of restricted feeding or fasting [28]. Astrocytes are the most abundant cell type in animal brains and play an important role in maintaining the function of the CNS [29]. The glial fibrillary acidic protein (GFPA) is considered a marker of mature astrocytes, and *PTGDS* is involved in regulating the synthesis of GFPA [30]. In the present study, heat stress leads to the down-regulation of the *PTGDS* gene, which may affect the maturation of astrocytes to a certain extent. *POMC* is well known to regulate feed intake via inducing anorexigenic and satiety in poultry [31,32]. In our previous study, we identified that heat stress leads to the mRNA expression of *POMC* genes being significantly up-regulated, which is consistent with RNA sequencing in our previous study [11]. Nitric oxide synthase (NOS) is an isoenzyme that exists in endothelial cells, macrophages, nerve phagocytes, and nerve cells. NOS, which exists in neurons, is selectively distributed in different brain regions [33]. Nitric oxide synthase 1 (*NOS1*) is produced in the nervous tissues of the CNS and peripheral nervous system and assists in cell communication and union with the proto-membrane [33,34]. Previous research has shown that oxidative stress down-regulates the *NOS1* gene significantly [35] and demonstrates that heat stress down-regulates the *NOS1* gene in broilers, suggesting that the expression level of the *NOS1* gene in animals is affected by either internal or external stress-related factors.

## 5. Conclusions

In the present study, we detected a total of 280 differential expression genes (HS vs. NC groups: 115 DEGs; PF vs. NC groups: 16 DEGs; and HS vs. PF groups: 149 DEGs) potentially associated with the digestion, absorption, and transport of nutrients, as well as appetite regulation via the hypothalamus transcriptome sequencing analysis of broilers. Furthermore, we validated seven key genes that may affect appetite, nutrient transportation, and neurotransmission: *SLC1A6*, *SLC6A13*, *SLC13A4*, *SLC16A8*, *PTGDS,* and *NOS1*. According to the GO and KEGG enrichment analysis, we initially explored one pathway that could affect protein digestion and absorption. These genes and pathways may play an important role in alleviating the adverse effects of decreased feed intake caused by heat stress.

## Figures and Tables

**Table 1:** DEGs among the three groups.

Treatment	HS_NC	PF_NC	HS_PF
Up-regulated genes	3	3	24
Down-regulated genes	112	13	125
Total genes	115	16	149

Note: NC, normal control group; HS, heat stress group; PF, pair feed group.

**Table 2:** The DEGs between the HS and NC groups.

Gene Name	H1	H2	H3	N1	N2	N3	Up/Down	*p*-Value
*SLC1A6*	291	385	394	881	1465	340	down	3.34 × 10^−9^
*NOS1*	210	237	286	359	969	235	down	7.64 × 10^−6^
*SLC6A13*	43	21	54	370	37	55	down	3.24 × 10^−4^
*SLC13A4*	22	8	13	820	35	8	down	2.50 × 10^−9^
*POMC*	363	199	388	68	108	54	up	3.77 × 10^−6^
*PTGDS*	3768	3454	4827	16,338	5252	5500	down	1.16 × 10^−8^
*SLC16A8*	2	3	3	38	68	9	down	1.56 × 10^−8^

Notes: *SLC1A6*, solute carrier family 1 member 6; *NOS1*, nitric oxide synthase 1; *SLC6A13*, solute carrier family 6 member 13; *SLC13A4*, solute carrier family 13 member 4; *POMC*, pro-opiomelanocortin; *PTGDS* stands for prostaglandin D2 synthase; *SLC16A8*, solute carrier family 16 member 8. H1, H2, H3 stands for heat stress group; N1, N2, N3 stands for normal group.

## Data Availability

The data presented in this study are available upon request from the corresponding author.

## Supplementary Materials

The following supporting information can be downloaded at: https://www.mdpi.com/article/10.3390/ani15040502/s1, Table S1. Sequences used for real-time PCR primers; Table S2. Information of reads in RNA-Seq.
