# Supplementary material for: Identification of Differentially Expressed Genes in the Hypothalamus of Broilers Under Heat Stress Using Transcriptome Analysis
Source: Animals (Basel). 2025 Feb 10;15(4):502. doi: 10.3390/ani15040502 (PMC11852300; doi:10.3390/ani15040502)
Supplement: Supplementary file 1 [file animals-15-00502-s001.zip › animals-3374027-supplementary.pdf]

Table S1 Sequences used for real-time PCR primers

Gene	Gene bank	Primer sequence 5’-3’	Product size
SLC16A8	NM_205140.2.	F: ATCTGCAGTGCCAGGGCTAG	115
		R: CAACGATGGCCATCAGCACC	
NOS1	XM_015294875.1	F: GCCAGCACGTCCGCAAGTC	137
		R: CACCTCCTCCAGCCTCTCCATG	
SLC13A4	XM_416360.4	F: TTCTCATTCCACTTCTGCTGCTTCC	134
		R: AATTAAGGCTGCTGCACCAAGAGG	
SLC6A13	XM_003640400.4	F: TTCTTCCTGGAGACGGCACTGG	113
		R: CAACGATGACCTGAGAGGCATAACC	
SLC1A6	XM_004948655.2	F: CAAGACGCAGTACAGCACCAGAG	126
		R: GTGACATTCTCCAGGATGCCAGTG	
PTGDS	NM_204259.1	F: GCCTCCAACTCCAACTGGTTCAAG	138
		R: GCTGTTCCTCTTCTCGCACTGTTC	
GAPDH	NM_204305.1	F: GAGGGTAGTGAAGGCTGCTG	113
		R: CATCAAAGGTGGAGGAATGG	

Table S2 Information of reads in RNA-Seq

Sample	N1	N2	N3	H1	H2
Raw Reads Number	56,122,658	51,874,886	51,947,322	51,549,182	53,360,734
Raw Bases Number	8,418,398,700	7,781,232,900	7,792,098,300	7,732,377,300	8,004,110,100
Clean Reads Number	46,012,946	43,762,562	43,020,862	42,659,330	44,205,794
Clean Reads Rate（%）	81.99	84.36	82.82	82.75	82.84
Clean Bases Number	6,901,941,900	6,564,384,300	6,453,129,300	6,398,899,500	6,630,869,100
Low-quality Reads Number	9,729,916	7,705,540	8,563,420	8,540,356	8,815,160
Low-quality Reads Rate（%）	17.34	14.85	16.48	16.57	16.52
Ns Reads Number	16	16	24	24	32
Ns Reads Rate（%）	0	0	0	0	0
Adapter Polluted Reads Number	379,780	406,768	363,016	349,472	339,748
Adapter Polluted Reads Rate（%）	0.68	0.78	0.7	0.68	0.64
Raw Q30 Bases Rate（%）	88.62	89.71	88.99	89	88.97
Clean Q30 Bases Rate（%）	94.21	94.43	94.29	94.32	94.28

Table 2 Information of reads in RNA-Seq（to continue）

Sample	H3	P1	P2	P3
Raw Reads Number	59,376,726	50,271,430	48,036,858	53,857,674
Raw Bases Number	8,906,508,900	7,540,714,500	7,205,528,700	8,078,651,100
Clean Reads Number	49,399,246	41,795,974	39,788,128	44,363,498
Clean Reads Rate（%）	83.2	83.14	82.83	82.37
Clean Bases Number	7,409,886,900	6,269,396,100	5,968,219,200	6,654,524,700
Low-quality Reads Number	9,508,120	8,153,114	7,950,642	9,105,944
Low-quality Reads Rate（%）	16.01	16.22	16.55	16.91
Ns Reads Number	40	268	30	20
Ns Reads Rate（%）	0	0	0	0
Adapter Polluted Reads Number	469,320	322,074	298,058	388,212
Adapter Polluted Reads Rate（%）	0.79	0.64	0.62	0.72
Raw Q30 Bases Rate（%）	89.18	89.29	88.93	88.84
Clean Q30 Bases Rate（%）	94.29	94.49	94.26	94.28
